# The vagus nerve as a neurovisceral interface: a comprehensive review

**DOI:** 10.3389/fnins.2026.1878765

**Published:** 2026-07-16

**Authors:** So-Young In, Tae-Hyeon Cho, Hun-Mu Yang

**Affiliations:** 1 Translational Laboratory for Clinical Anatomy, Department of Anatomy, Yonsei University College of Medicine, Seoul, Republic of Korea; 2 Department of Anatomy, Wonkwang University School of Medicine, Iksan, Republic of Korea; 3 Institute of Wonkwang Medical Science, Wonkwang University, Iksan, Republic of Korea

**Keywords:** autonomic system, cardiovascular system, gut-brain axis, vagus nerve, vagus nerve stimulation

## Abstract

The vagus nerve is the longest cranial nerve and a key component of the autonomic nervous system, functioning as a neurovisceral interface between the brain and peripheral organs. Despite well-defined anatomy, the mechanisms underlying its integrative roles in cardiovascular, metabolic, and neuropsychiatric regulation remain incompletely understood. This narrative review synthesizes current evidence on the anatomical organization, physiological functions, and clinical relevance of the vagus nerve, focusing on cardiac autonomic control, gastrointestinal and metabolic regulation, the gut–brain axis, and vagus nerve stimulation. In the cardiovascular system, it interacts with sympathetic pathways within the cardiac plexus and intrinsic cardiac nervous system to regulate heart rate and conduction. In the gastrointestinal system, it coordinates motility, secretion, and metabolic homeostasis through nutrient- and hormone-sensitive pathways. Within the gut–brain axis, emerging evidence highlights rapid neuroepithelial signaling and microbiota-dependent modulation mediated by vagal circuits. The vagus nerve stimulation represents a promising therapeutic strategy for restoring autonomic balance, although challenges remain in fiber selectivity and clinical variability. Advances in multi-omics approaches are beginning to reveal the molecular heterogeneity of vagal neurons, but significant gaps persist due to limited human anatomical data. In conclusion, the vagus nerve functions as a multidimensional integrative system, and a deeper understanding of its structure and molecular organization is essential for developing precise neuromodulatory therapies.

## 1 Introduction

**Figure 1:**
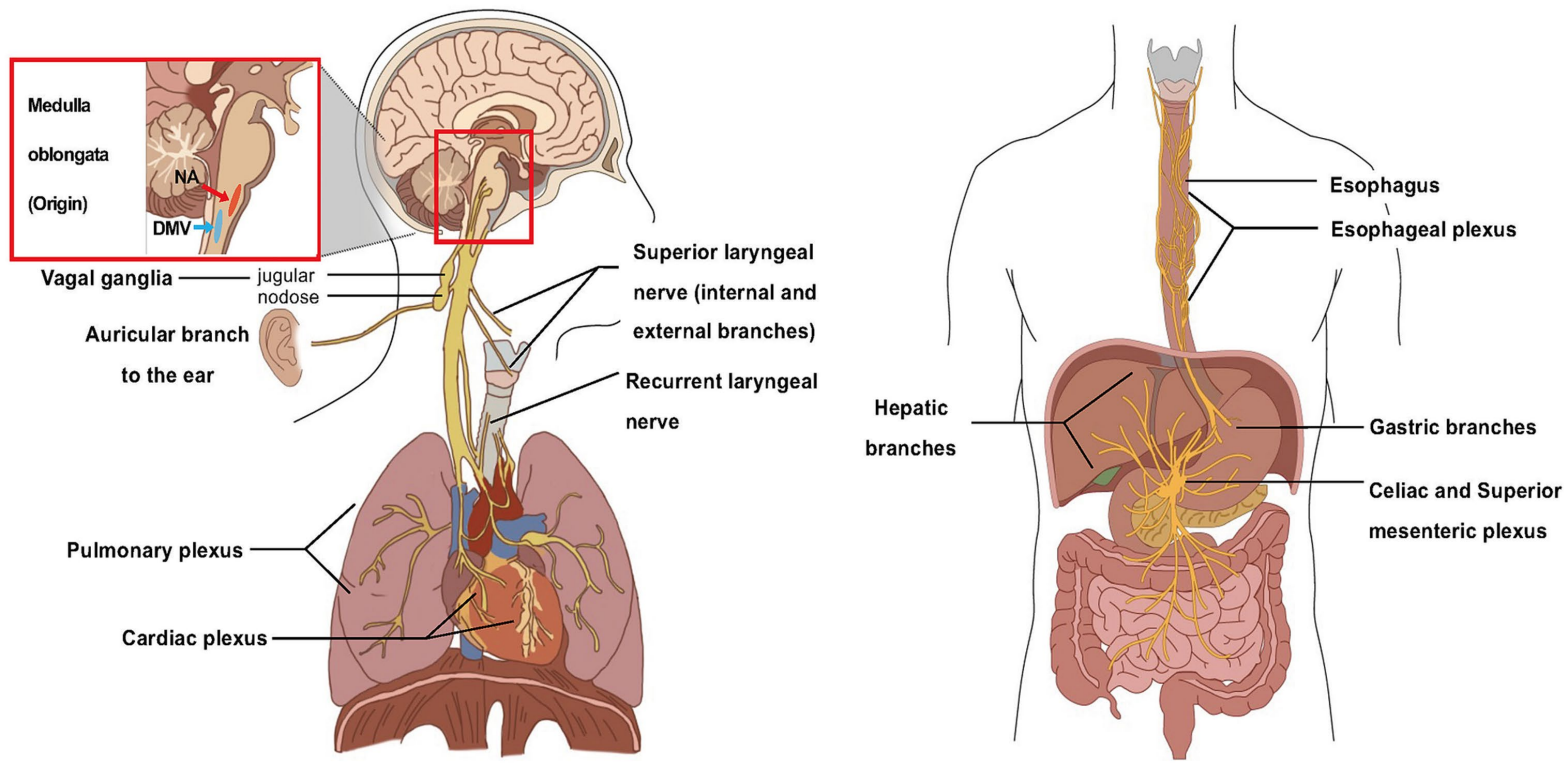
Anatomy of the sensory vagus nerve. It depicts the vagus nerve and its major distribution from the medulla oblongata through the head and neck into the thorax and abdomen, showing branches to the ear, larynx, heart, lungs, esophagus, liver, stomach, intestine, and pancreas.

The vagus nerve (CN X) is the longest and most complex cranial nerve, serving as the fundamental axis of the autonomic nervous system. Anatomically, it originates from multiple specialized nuclei in the medulla oblongata, including the dorsal motor nucleus of vagus nerve (DNV), the nucleus ambiguous (NA), and the nucleus tractus solitarius (NTS) (Berthoud and Neuhuber, 2000; Nieuwenhuys et al., 2008; Standring, 2021; Prescott and Liberles, 2022). Upon emerging from the lateral medulla, the nerve exits the cranium through the jugular foramen and descends through the neck within the carotid sheath. As it traverses the cervical region, it gives off -vital branches such as the superior and recurrent laryngeal nerves, which are essential for laryngeal function and airway protection. This pathway continues through the mediastinum of the thorax, forming the esophageal plexus and reaches the abdominal cavity. This structural continuity allows the vagus nerve to act as a principal bidirectional conduit, facilitating rapid communication between the brain, the heart, and the abdominal viscera (Berthoud and Neuhuber, 2000; Nieuwenhuys et al., 2008; Standring, 2021; Prescott and Liberles, 2022) (Figure 1).

As the primary mediator of the parasympathetic nervous system, the vagus nerve provides direct innervation to target organs through a mix of efferent and afferent fibers (Berthoud and Neuhuber, 2000; Nieuwenhuys et al., 2008; Standring, 2021; Prescott and Liberles, 2022). In the cardiovascular system, vagal efferents exert crucial control over heart rate and conduction velocity, while sensory afferents provide real-time feedback by monitoring cardiac muscle status and baroreceptor signals (Bender et al., 2026; Liu et al., 2026). In its abdominal course, the nerve governs metabolic homeostasis and the gut-brain axis. It not only regulates gastrointestinal motility and secretion but also conveys gut-derived signals that may contribute to affective and behavioral regulation through central interoceptive pathways (Siopi et al., 2023; Longo et al., 2023; Décarie-Spain et al., 2024). These diverse functions highlight the nerve’s role as a critical interface between the body’s internal environment and the central nervous system.

Despite the clinical importance of these pathways, the full extent of the vagus nerve’s functional mechanisms and its precise axonal composition remain insufficiently understood. While the general anatomy is well-established, current research is still uncovering the specific ways in which vagal signaling modulates complex physiological and psychological processes (Biscola et al., 2024; Chen and Liu, 2025; Thompson et al., 2026). Nevertheless, some unresolved questions remain regarding organ-specific vagal organization, circuit-level functional organization, and the translational gap between animal models and humans. Therefore, this review provides a comprehensive analysis of the latest findings regarding the vagus nerve, focusing on its cardiac regulatory mechanisms and its metabolic and psychological roles within the gut-brain axis. By synthesizing these current insights, this paper aims to clarify the known roles of the vagus nerve in these systems and identify areas where further research is needed.

## 2 Meterials and methods

### 2.1 Literature search and selection criteria

This narrative review was conducted to synthesize current evidence on the anatomical organization, physiological roles, and clinical relevance of the vagus nerve, with particular emphasis on its cardiovascular regulation, gastrointestinal and metabolic functions, gut–brain signaling, and therapeutic modulation through vagus nerve stimulation.

A comprehensive literature search was performed using PubMed, Scopus, and Web of Science databases. The search strategy combined Medical Subject Headings (MeSH) and free-text terms related to vagal anatomy, autonomic regulation, cardiac control, gastrointestinal physiology, metabolic signaling, gut–brain communication, and neuromodulation. Search terms included “vagus nerve,” “cranial nerve X,” “cardiac autonomic nervous system,” “cardiac plexus,” “intrinsic cardiac nervous system,” “ganglionated plexus,” “vagal afferents,” “vagal efferents,” “nucleus tractus solitarius,” “dorsal motor nucleus of the vagus nerve,” “nucleus ambiguus,” “gut–brain axis,” “vagal gastrointestinal signaling,” “metabolic regulation,” “vagus nerve stimulation,” “transcutaneous auricular vagus nerve stimulation,” “transcutaneous cervical vagus nerve stimulation,” “single-cell RNA sequencing,” and “spatial transcriptomics.”

Studies were screened based on their relevance to the structural and functional organization of the vagus nerve. Particular attention was given to articles describing vagal pathways in cardiovascular regulation, gastrointestinal and metabolic homeostasis, vagal sensory mechanisms, neuroimmune signaling, and recent methodological advances in vagal neurobiology.

### 2.2 Inclusion and exclusion criteria

Studies were included if they met the following criteria:

1. Published in peer-reviewed journals through 2026.
2. Written in English.
3. Addressed the anatomy, physiology, molecular organization, or clinical modulation of the vagus nerve.
4. Investigated vagal involvement in cardiac regulation, gastrointestinal function, metabolic control, gut–brain communication, or inflammatory signaling.
5. Included original research articles, review articles, systematic reviews, meta-analyses, or major translational studies relevant to vagal function.

Studies were excluded if they:

1. Focused on autonomic regulation without specific reference to the vagus nerve.
2. Discussed cardiovascular, gastrointestinal, metabolic, or psychiatric disease without mechanistic relevance to vagal pathways.
3. Were limited to peripheral stimulation techniques without addressing anatomical or neurophysiological mechanisms.
4. Were conference abstracts, editorials, opinion pieces, letters, or non-peer-reviewed publications.
5. Were not available in full text or were not written in English.

Reference lists of selected articles and recent review papers were manually examined to identify additional relevant studies not captured through the initial database search.

### 2.3 Data extraction and thematic categorization

Relevant information was extracted from each selected article, including anatomical structures, neural pathways, physiological mechanisms, molecular markers, disease-related alterations, and therapeutic implications. Extracted data were organized into the following thematic categories:

1. The Vagal Architecture of Heart Control.
2. Vagal Regulation of Gastrointestinal and Metabolic Systems.
3. Gut–brain axis and neuroepithelial signaling.
4. Vagus nerve stimulation and therapeutic mechanisms.
5. Recent advances in omics, sequencing, neurotransmitter mapping, and human anatomical limitations.

The review was structured according to these categories to integrate anatomical, physiological, molecular, and clinical findings. This approach was intended to clarify the role of the vagus nerve as a bidirectional regulatory interface linking the brain, heart, gastrointestinal tract, immune system, and metabolic networks.

## 3 Results

### 3.1 The vagal architecture of heart control

#### 3.1.1 Structural and functional organization of the cardiac autonomic nervous system

Postganglionic sympathetic and preganglionic vagal fibers converge within the cardiac plexus - a mediastinal structure organized into superficial and deep layers - before branching into specialized epicardial, myocardial, and coronary perivascular plexuses. Contradicting classical views of segregated pathways, vagal and sympathetic nerves intermingle as early as the cervical level to form mixed nerve trunks (Palma and Benarroch, 2014). These trunks act as an integrated hub, suggesting that focal interventions on specific branches elicit complex, synergistic autonomic responses rather than sequestered effects. This high degree of inter-cervical and intrathoracic cross-talk implies that autonomic input to the heart is not a simple dualistic “push-pull” system, but rather a pre-integrated signal modulated before reaching the myocardium.

The cardiac plexus exhibits a lateralized organization with significant clinical implications. Anatomically, the right vagal pathway primarily modulates chronotropy via projections to the sinoatrial (SA) node, while the left pathway distributes to the atrioventricular (AV) node to modulate dromotropy and ventricular contractility (Zandstra et al., 2021). This asymmetry is mirrored in the sympathetic system: left stellate ganglion stimulation preferentially increases left ventricular contractility and blood pressure, whereas right stellate ganglion stimulation primarily affects heart rate (Ajijola et al., 2015). However, recent human and porcine studies have challenged this functional laterality, finding no significant asymmetry during vagal activation (Yamakawa et al., 2014; Sharma et al., 2025). Consistent with these findings, both right and left VNS produced similar ventricular electrophysiological effects in the porcine model, suggesting that convergence within intrinsic cardiac neural networks may attenuate side-specific differences and distribute vagal effects more broadly across the ventricular myocardium (Yamakawa et al., 2014). This suggests that the cardiac plexus functions as an integration hub that homogenizes side-specific effects through complex neural networking. Consequently, this structural “smearing” of functional laterality necessitates a more nuanced approach to targeted neuromodulation, as the cardiac plexus may effectively redistribute side-specific therapeutic stimuli across the entire intracardiac network, potentially diluting intended site-specific responses while minimizing unilateral adverse effects.

#### 3.1.2 The epicardial plexus: a ‘little brain’ coordinating cardiac function

Cardiac autonomic control arises from the sophisticated interplay between extracardiac circuits and the intrinsic cardiac nervous system (ICNS). The epicardial plexus, the primary framework of the ICNS, consists of ganglionated plexi (GP) embedded within epicardial fat pads (Armour, 2008). These GPs form a network of approximately 1,500 ganglia and 43,000 neurons, which are increasingly viewed as a single functional unit due to their extensive interconnections (Armour et al., 1997; Pauza et al., 2002). Regional classifications vary: Armour et al. (1997) defined 10 distinct GPs in human heart, while Pauza et al. (2002) identified 7 subplexuses based on anatomical pathways in the dog heart. Regardless of the classification used, the epicardial plexus operates as an integrated network, explaining why localized GP ablation or stimulation often produces broad and complex physiological responses (Katritsis et al., 2013).

Driven by local circuit neurons, the ICNS functions as a “little brain,” processing and exchanging information independently to coordinate cardiac function (Ardell and Armour, 2016). GPs contain diverse populations of afferent, efferent, and local circuit neurons (Palma and Benarroch, 2014). These populations integrate into three major mixed nerves - the left coronary, left lateral, and right coronary cardiac nerves - which course along the coronary arteries to supply both the atria and ventricles (Gumpangseth et al., 2019). Mapping studies using tyrosine hydroxylase (TH) and choline acetyltransferase (ChAT) markers show regional dominance: the right atrium possesses a high density of cholinergic (parasympathetic) fibers for heart rate regulation, while the ventricles and left atrium are predominantly under adrenergic (sympathetic) control (Hoover et al., 2004; Fedele and Brand, 2020). However, a clear consensus on regional fiber density and neurochemical profiles in the human heart remains elusive (Nerantzis et al., 1996; Kawano et al., 2003).

#### 3.1.3 The right atrial Ganglionated plexus (RAGP): a vagal orchestration center

Vagal innervation mediates negative chronotropic, dromotropic, and inotropic effects. Traditionally, right vagal fibers were thought to target the SA node exclusively via the RAGP. However, recent research has demonstrated that the RAGP also serves as a primary site for modulating AV conduction (Hanna et al., 2017; Hanna et al., 2021). Anatomically, the RAGP comprises superior and inferior components located near the superior vena cava-right atrium (SVC-RA) junction and the interatrial groove, respectively (Armour et al., 1997). RAGP neurons exhibit remarkable neurochemical diversity, co-expressing ChAT and TH to enable dual modulation of nodal activities (Hanna et al., 2021). Porcine models reveal that the RAGP integrates signals from both vagal nerves to modulate the SA node, AV node, and left ventricular contractility, further challenging the paradigm of exclusive right vagal influence on the SA node (Hanna et al., 2021). While 99% of RAGP neurons are cholinergic, they show significant phenotypic diversity, with 13% expressing TH and 84% expressing neuropeptide Y (NPY). Their structural organization - synapses concentrated on dendrites rather than the soma - supports a synaptic convergence system for integrating multiple neural inputs. Based on recent experimental study from porcine models, the RAGP has been proposed to function as a “pre-nodal processor,” filtering and fine-tuning bilateral vagal inputs before they reach the cardiac conduction system (Hanna et al., 2021). Consequently, the RAGP acts as a critical gateway that coordinates synchronization between atrial rate and atrioventricular conduction. Beyond simple signal transmission, this site-specific neurochemical heterogeneity - particularly the presence of NPY and TH - suggests that the RAGP can dynamically shift its regulatory output in response to fluctuating autonomic demands, potentially acting as a local buffer against extreme vagal surges or sympathetic overactivity. As 21–24% of porcine RAGP neurons project to the SA node, they exert a decisive influence on electrical activation. Ultimately, functionally specific targeting of the RAGP - aimed at modulating its integrative capacity rather than inducing total denervation - is essential to optimize clinical outcomes and preserve physiological rate-responsiveness in atrial fibrillation therapy (Hanna et al., 2021).

#### 3.1.4 Ventricular autonomic innervation and pathophysiological remodeling

Ventricular innervation patterns reflect specific hemodynamic roles. The right ventricle is governed by right sympathetic and vagal nerves, with a high fiber density in the right ventricular outflow tract (RVOT). This anatomical concentration makes the RVOT a common origin for arrhythmias (Hanna et al., 2021). In contrast, the left ventricle features a complex reticular network of fibers penetrating the deep myocardium to support contractility, largely regulated by the left stellate ganglion (Hanna et al., 2021; Tompkins et al., 2025). In pathological states, the autonomic system undergoes profound structural and neurochemical remodeling. Atrial fibrillation is associated with neuronal nitric oxide synthase (nNOS)/ChAT imbalances and ganglionic hyperexcitability (Hoover et al., 2004). Myocardial infarction (MI) causes localized denervation and abnormal sympathetic sprouting in the peri-infarct zone, driving fatal ventricular arrhythmias. Heart failure results in reduced RAGP activity, loss of SA node control, and phenotypic remodeling of neuronal somata and synaptic efficiency (Hoover et al., 2004; Hanna et al., 2021; Tompkins et al., 2025). Critically, this neuroplasticity often involves a “phenotypic switch” where neurons shift their neurotransmitter expression, further impairing the ICNS’s ability to stabilize cardiac rhythm. These maladaptive changes transform the once-protective “little brain” into a pro-arrhythmic substrate, exacerbating autonomic dysregulation in a vicious cycle (Ardell and Armour, 2016).

#### 3.1.5 The vagal sensory system: monitoring cardiac homeostasis

**Figure 2:**
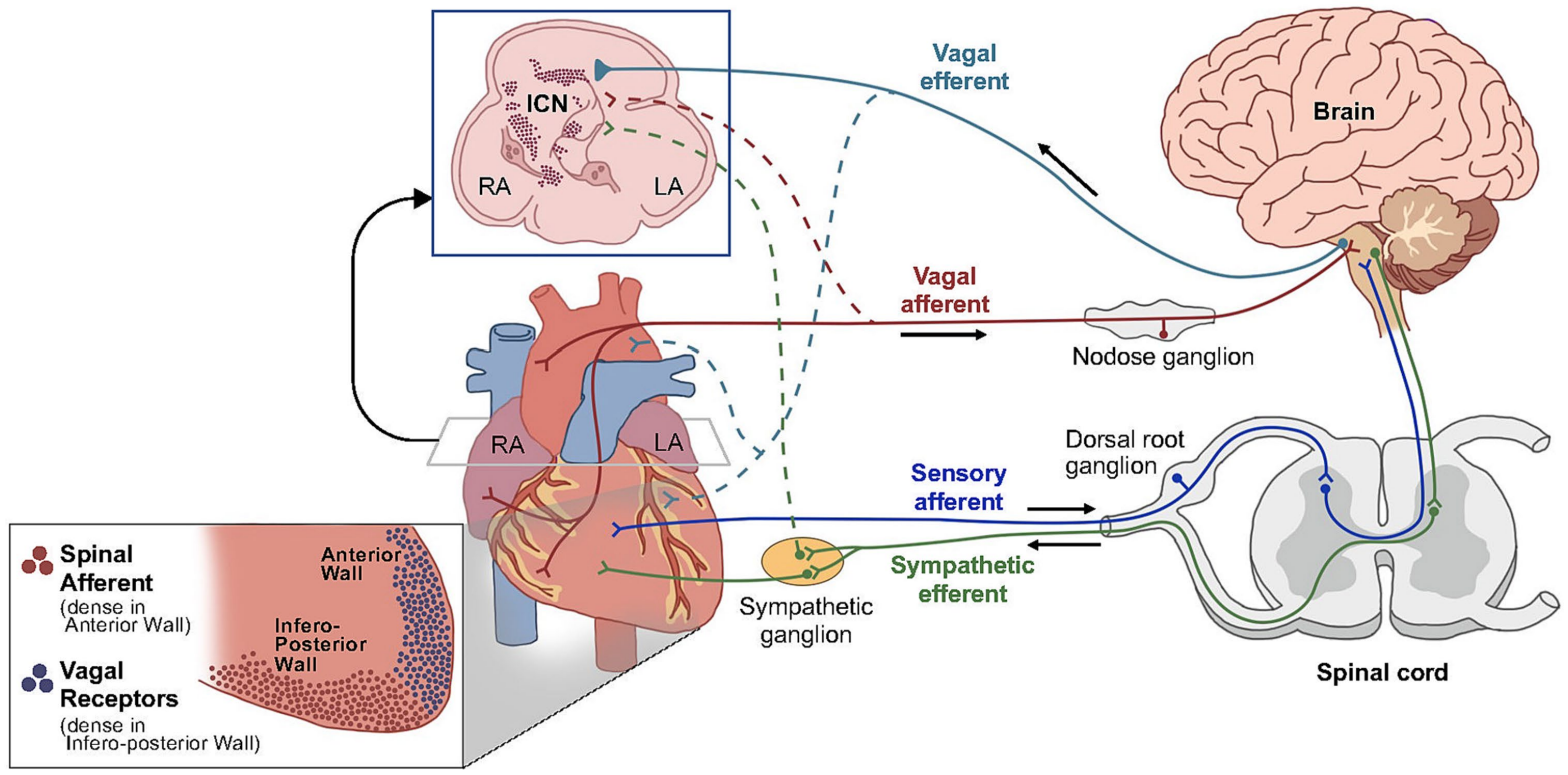
Structural and functional organization of the heart-brain circuit. The Heart-Brain circuit functions as an integrated hub where sympathetic and vagal pathways converge to pre-process cardiac signals. Spinal afferents (dense in the anterior wall of the left ventricle) and vagal afferents (dense in the infero-posterior wall of the left ventricle) relay to the central nervous system, while the intrinsic cardiac nervous system (ICNS) serves as a “little brain” within the epicardial plexus to fine-tune autonomic output. The RA and LA means the right and left atrium. This figure is modified from Mohanta et al. (2023). Source: Figure 3. License: American Heart Association (permission not yet obtained). Material: Adapted (redrawn with additional original content).

The heart is innervated by two primary visceral sensory pathways: vagal afferents, which enter the nodose ganglion and trigger parasympathetic responses, and spinal afferents, which relay through the spinal cord to induce sympathetic reflexes (Malliani, 2000; Jänig, 2022). Vagal receptors are concentrated in the infero-posterior wall of the left ventricle (Thorén, 2005; Shenton et al., 2021), while spinal afferents are most dense in the anterior wall (Minisi and Thames, 1991). This structural arrangement, highlighted in Figure 2, illustrates the distinct spatial organization of afferent pathways across the cardiac landscape (Mohanta et al., 2023). In rodent model, vagal sensory receptors include mechanoreceptors, which detect myocardial deformation via channels such as PIEZO1, PIEZO2, TRPC5, ASIC2, and Tentonin3 (Zeng et al., 2018; Min et al., 2019), and chemoreceptors (C-fibers) that respond to metabolic and inflammatory substances like reactive oxygen species (ROS). Transient Receptor Potential Vanilloid 1 (TRPV1)-positive nociceptors release Calcitonin Gene-Related Peptide (CGRP) and Substance P to mediate cardioprotective reflexes and pain (Kupari et al., 2019). While 80% of vagal sensory neurons serve the gastrointestinal tract, only 5–6% are dedicated to cardiac information (Berthoud and Neuhuber, 2000). The nodose ganglion lacks distinct viscerotopic organization (Altschuler et al., 1989; Zhuo et al., 1997), but the NTS in the brainstem possesses a topographical structure, with the dorsomedial part leading cardiovascular reflexes (Kalia and Sullivan, 1982; Thayer and Koenig, 2025). Beyond projections to the NA and DMV, ascending NTS pathways also engage parabrachial, hypothalamic, and limbic regions, providing a route through which vagal sensory information may influence behavioral and affective regulation (Saper, 2002; Holt, 2022). Glutamate is the primary neurotransmitter; specifically, binding to N-methyl-D-aspartate (NMDA) receptors induces bradycardia, whereas binding to non-NMDA receptors triggers a rise in arterial pressure (Song and Zhao, 1993). Gamma-aminobutyric acid (GABA) and dopamine also modulate blood pressure (Song and Zhao, 1993), while nitric oxide (NO) acts as an inhibitory signal to lower heart rate and pressure (Harada et al., 1993).

In disease states, vagal efferent tone decreases due to insufficient afferent input and increased central inhibition (Schwartz et al., 1988). Myocardial infarction induces denervation and abnormal sprouting (Cao et al., 2000). In a porcine model of chronic myocardial infarction, cardiac nociceptive stimulation produced predominantly inhibitory responses in nodose ganglion neurons, accompanied by increased GABAergic marker expression in CGRP-positive nociceptive neurons (Salavatian et al., 2022). Mitochondrial dysfunction and oxidative stress further impair voltage-gated sodium channels and afferent function (Zhang et al., 2014). Furthermore, hypertensive states weaken mechanotransduction by reducing PIEZO2 expression (Zeng et al., 2018). These combined structural and molecular changes disrupt the critical cardioprotective feedback loop, transforming the once-stabilizing afferent system into a driver of autonomic dysregulation. These pathological alterations across multiple domains are summarized in Table 1.

**Table 1:** Summary of cardiac vagal afferent signaling in health and disease.

Comparison	Healthy state	Disease state
Overall vagal afferent signaling	Continuous transmission of cardiac sensory information via vagal afferents	Reduced vagal afferent signaling and impaired transduction
Autonomic balance	Reflex parasympathetic facilitation and cardiac autonomic fine-tuning	Sympathovagal imbalance, increased sympathetic tone, and parasympathetic withdrawal
Chemosensitive/nociceptive signaling	TRPV1-positive nociceptive afferent activity in response to chemical stimuli	Paradoxical reduction of functional nociceptive signaling, with increased CGRP/GABA coexpression
Mechanosensitive signaling	PIEZO2-dependent mechanotransduction of myocardial stretch and pressure	Decreased PIEZO2-positive mechanosensitive neurons and diminished mechanotransduction
Structural/metabolic changes	Intact neuronal function and preserved afferent population	Denervation, peri-infarct sprouting, GFAP increase, and metabolic/oxidative dysfunction
Clinical consequences	Support of parasympathetic reflex control and cardiac homeostasis	Heart failure progression, ventricular arrhythmia susceptibility, and increased sudden death risk

The table highlights the preserved transmission of cardiac sensory information and reflex parasympathetic control under healthy conditions, versus the reduced afferent signaling, sympathovagal imbalance, paradoxical alteration of chemosensitive and mechanosensitive transduction, structural remodeling, and adverse clinical consequences observed in disease states. Adapted from van Weperen and Vaseghi (2023).

Current research is hindered by the anatomical inaccessibility of the human nodose ganglion (Settell et al., 2020), leading to a significant gap in transcriptomics data and difficulty in projecting rodent findings (Kupari et al., 2019) to human physiology. This lack of human data remains a primary barrier to discovering specific markers and elucidating the molecular remodeling underlying arrhythmia and other disease states (van Weperen and Vaseghi, 2023).

### 3.2 Vagal regulation of gastrointestinal and metabolic systems

#### 3.2.1 Vagal gastrointestinal structural-functional framework

The vagus nerve establishes its regulatory reach through a precise peripheral course, with its thoracic and abdominal branches providing the structural foundation for gastrointestinal control (Berthoud and Neuhuber, 2000; Browning and Travagli, 2014; Travagli and Anselmi, 2016). Within the mediastinum, the right and left nerves converge to form the esophageal plexus, a complex network that ensures the primary peristaltic wave through the smooth muscle layers (Berthoud and Neuhuber, 2000). Upon reaching the diaphragm, these fibers reorganize into the anterior and posterior vagal trunks, passing through the esophageal hiatus to enter the abdominal cavity. The distribution of these trunks marks a transition from localized motility to broad systemic integration (Berthoud and Neuhuber, 2000; Browning and Travagli, 2014; Travagli and Anselmi, 2016). The anterior trunk distributes branches to the stomach and liver, while the posterior trunk extends its influence through the celiac plexus to innervate a vast portion of the midgut (Berthoud and Neuhuber, 2000; Furness, 2012; Travagli and Anselmi, 2016). This peripheral branching is not merely motor-centric; for instance, the hepatic branches form a direct neural link with the hypothalamus creating a structural basis for a neuro-metabolic axis (Berthoud, 2008; Grill and Hayes, 2012). By integrating with sympathetic fibers, the thoracic and abdominal vagus nerve operates as a mixed-nerve interface, providing the essential anatomical framework for the high-dimensional metabolic and sensory integration that governs systemic homeostasis (Furness, 2012; Travagli and Anselmi, 2016).

The vagus nerve operates as a sophisticated regulatory axis where peripheral sensory inputs from the esophagus, stomach, intestine, and accessory digestive organs project to the NTS, while efferents from DMV and NA govern motility, secretion, and essential vagovagal reflexes (Travagli et al., 2006; Browning and Travagli, 2010; Bonaz et al., 2017). Beyond classical autonomic efferent control, recent studies in mice have identified molecularly and functionally distinct vagal afferent neuron (VAN) subtypes responsive to mechanical stretch, nutrients, and inflammatory mediators (Williams et al., 2016; Bai et al., 2019). This specialization allows the vagus nerve to convey precise interoceptive information through dedicated channels rather than generalized signals.

Consequently, gastrointestinal parasympathetic regulation is functionally inseparable from visceral sensing; the same neural architecture coordinates the transition from ingestion to digestion while simultaneously informing the central nervous system (CNS) of the physiological conditions required to dynamically adjust those responses (Travagli et al., 2006; Bonaz et al., 2017). Thus, vagal signaling is a critical determinant in disorders such as functional dyspepsia and nausea, where symptoms arise from altered sensorimotor integration rather than isolated structural lesions (Tack et al., 2006; Van oudenhove et al., 2016). This integrative framework explains why disrupted parasympathetic tone often manifests as overlapping gastrointestinal symptoms—impairing accommodation, motility, and sensation simultaneously (Tack et al., 2006). Ultimately, the vagus nerve acts as the primary neural interface between the gut and the CNS, linking fundamental digestive physiology with complex, symptom-based disorders interpreted through gut-brain mechanisms (Travagli et al., 2006; Browning and Travagli, 2010).

Given the vagus nerve’ role as the fundamental structural link between the CNS and the gut, vagotomy (the severance of the vagus nerve) is anatomically grounded in its ability to suppress parasympathetic overactivity (Dragstedt, 1945; Schirmer, 1989; Debas and Carvajal, 1994). Its primary clinical utility was established in the surgical management of peptic ulcer disease, where severing the overactive vagal pathways counteracts excessive gastric acid secretion (Dragstedt, 1945). By interrupting the cephalic phase of digestion, this procedure effectively reduces parietal cell stimulation and lowers the risk of ulcer recurrence (Debas and Carvajal, 1994). The application of vagotomy is rooted in the recognition of the vagus nerve as the master regulator of gastric physiology (Browning and Travagli, 2010). Because the vagal trunks consist of mixed fibers, surgical interruption not only suppresses acid but also dismantles the sensory and reflex circuits essential for coordinated gastric function (Browning and Travagli, 2010; Bonaz et al., 2017). Crucially, vagotomy serves as a functional model of gut-brain disconnection, representing the severance of a bidirectional information channel rather than the mere denervation of an effector organ. This reframes the procedure beyond its antisecretory intent, as interrupting vagal continuity results in system-level consequences that extend far beyond the targeted suppression of parietal cells.

#### 3.2.2 Metabolic regulation and dysfunctions of the vagal system

Recent research has expanded the role of the vagus nerve from classical gastrointestinal control to a master regulator of metabolic homeostasis (Dockray, 2009; Waise et al., 2018; Bai et al., 2019; Wachsmuth et al., 2022). VANs are strategically positioned to detect not only mechanical distension but also nutrient-derived and hormone-mediated signals (Dockray, 2009; Waise et al., 2018; Williams et al., 2016; Bai et al., 2019;). Serving as a sophisticated sensory interface, these neurons inform the brain of ingested nutrients to regulate appetite, meal size, hepatic glucose production, insulin sensitivity, and whole-body glucose homeostasis (Dockray, 2014; Waise et al., 2018; Wachsmuth et al., 2022). This metabolic relay is mediated by peripheral signals such as cholecystokinin (CCK), glucagon-like peptide-1 (GLP-1), peptide YY (PYY), and ghrelin (Date et al., 2002; Murphy and Bloom, 2006; Dockray, 2009; Waise et al., 2018). The metabotropic receptors through which these signals act on vagal afferent neurons, along with their appetite-related effects, are summarized in Table 2. Notably, rodent studies showing that ghrelin-induced feeding and growth hormone secretion depend heavily on gastric vagal afferents illustrates how the vagus nerve links luminal events to central feeding programs (Date et al., 2002). Beyond nutrient sensing, the vagus nerve serves as a neuroimmune-metabolic interface. The “inflammatory reflex” has established that the nervous system regulates real-time inflammation through vagal pathways (Tracey, 2002; Pavlov and Tracey, 2012). In conditions of metabolic dysfunction, vagal signaling influences inflammatory tone—a critical factor given that chronic low-grade inflammation is a primary driver of insulin resistance and Type 2 Diabetes Mellitus (T2DM) (Tracey, 2002; Pavlov and Tracey, 2012).

**Table 2:** Metabotropic G protein-coupled receptors expressed in vagal afferents and their effects on appetite.

Receptor	Natural ligand	Effect on appetite	Species	References
CCK1R	CCK	Decrease	Mouse	Murphy and Bloom (2006), Berthoud (2008), Dockray (2009), Mayer (2011), Grill and Hayes (2012), Dockray (2014), de Lartigue (2016), Williams et al. (2016), Kaelberer et al. (2018), Waise et al. (2018), Bai et al. (2019), and Wachsmuth et al. (2022)
			Human	Murphy and Bloom (2006), Berthoud (2008), Dockray (2009), Mayer (2011), Grill and Hayes (2012), Dockray (2014), Waise et al. (2018), and Wachsmuth et al. (2022)
GLP1R	GLP1	Decrease	Mouse	Murphy and Bloom (2006), Berthoud (2008), Grill and Hayes (2012), Dockray (2014), de Lartigue (2016), Williams et al. (2016), Waise et al. (2018), Bai et al. (2019), Bellono et al. (2017), and Wachsmuth et al. (2022)
			Human	Murphy and Bloom (2006), Berthoud (2008), Grill and Hayes (2012), Waise et al. (2018), and Wachsmuth et al. (2022)
GHSR	Ghrelin	Increase	Mouse	Murphy and Bloom (2006), Berthoud (2008), Grill and Hayes (2012), Dockray (2014), de Lartigue (2016), Waise et al. (2018), and Wachsmuth et al. (2022)
			Human	Murphy and Bloom (2006), Berthoud (2008), Grill and Hayes (2012), Dockray (2014), Waise et al. (2018), Wachsmuth et al. (2022)
NPY2R	PYY	Decrease	Mouse	Murphy and Bloom (2006), Berthoud (2008), Grill and Hayes (2012), Dockray (2014), de Lartigue (2016), Waise et al. (2018), and Wachsmuth et al. (2022)
			Human	Murphy and Bloom (2006), Berthoud (2008), Grill and Hayes (2012), Dockray (2014), Waise et al. (2018), and Wachsmuth et al. (2022)

The table summarizes metabotropic G protein-coupled receptors identified on vagal afferent neurons, focusing on mouse and human species, along with their corresponding natural ligands and effects on appetite regulation. The table is modified from Waise et al. (2018). Source: Table 1. License: Springer Nature (permission not yet obtained). Material: Adapted (rewritten with additional original content).

The integrative role of the vagus nerve is central to the pathogenesis of T2DM and obesity (Tracey, 2002; Pavlov and Tracey, 2012; de Lartigue, 2016; Bonaz et al., 2017). Because the vagus nerve encodes nutrient availability, satiation, and post-ingestive reinforcement, any impairment in vagal signaling can lead to excessive caloric intake, altered food choice, dysregulated hepatic glucose production, and reduced insulin responsiveness (Berthoud, 2008; de Lartigue, 2016; Wachsmuth et al., 2022). This link between gastrointestinal sensing and metabolic disease suggests that impaired vagal gut–brain signaling may contribute to metabolic dysfunction; however, whether it acts as a primary driver or develops secondarily to obesity-associated metabolic and inflammatory changes remains context-dependent (Berthoud, 2008; de Lartigue, 2016; Wachsmuth et al., 2022). Supporting this pathogenic model, a wide array of obesity-related receptors—including those for leptin, CCK, and various gut-derived peptides—are expressed directly on VANs (Murphy and Bloom, 2006; Dockray, 2009; de Lartigue, 2016). The presence of these receptors enables VANs to detect signals related to the body’s energy status and adiposity signals (Dockray, 2009; de Lartigue, 2016). Modern circuit studies in mice further reinforce the vagus nerve’s role in governing both homeostatic and hedonic feeding behaviors: while intestinal sugar activates vagal-brainstem circuits to generate sugar preference, fat similarly drives fat preference through dedicated gut-brain pathways (Han et al., 2018; Tan et al., 2020). Consequently, vagal dysfunction in metabolic disease alters not only meal termination but also the wnutrient-specific reinforcement that drives overconsumption. Disease-associated vagal dysfunction arises from both peripheral and central mechanisms rather than a simple reduction in vagal tone. Metabolic and inflammatory disturbances can alter vagal afferent sensitivity and central processing within the NTS, with context-dependent effects on afferent and efferent pathways. This bidirectional remodeling may create a feedback loop that exacerbates metabolic dysfunction (Berthoud and Neuhuber, 2000; Pavlov and Tracey, 2012).

#### 3.2.3 Vagal gut-brain axis: neuroepithelial signaling and multidimensional integration

**Figure 3:**
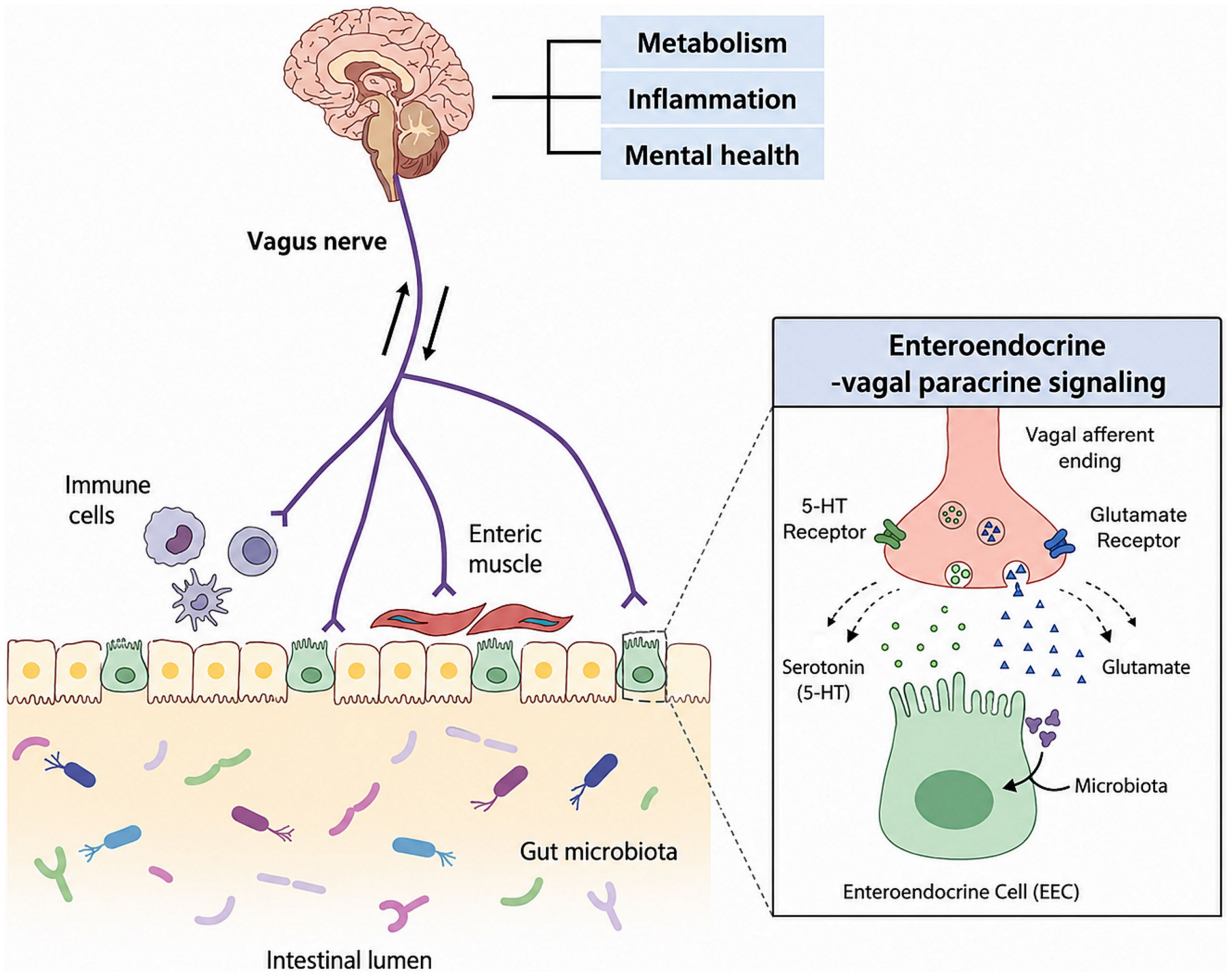
Mechanisms of gut-brain communication via the vagus nerve. The vagus nerve serves as a bidirectional conduit linking the gut to the brain, modulating systemic metabolism, inflammation, and mental health. Within the intestinal mucosa, enteroendocrine cells (EECs) respond to luminal stimuli and signals from the gut microbiota by releasing mediators such as serotonin and glutamate. These substances communicate with neighboring vagal afferent endings through paracrine, rather than synaptic, mechanisms, thereby contributing to gut–brain signaling and central autonomic responses. The figure is modified from Cryan and Dinan (2012). Source: Figure 1. License: Springer Nature (permission not yet obtained). Material: Adapted (redrawn with additional original content).

The contemporary view of the vagus nerve has evolved from a simple bridge between the CNS and peripheral organs into a sophisticated interface for high-dimensional interactions (Mayer, 2011; Browning and Travagli, 2014). Within the brain-gut axis—a complex, bidirectional network involving neural, endocrine, immune, and microbial pathways—vagal pathways provide a major neural route for conveying complex gut-derived information to central integrative circuits (Mayer, 2011; Cryan and Dinan, 2012). This framework is anchored in Mayer’s model, which posits that gastrointestinal homeostasis depends on continuous signaling between the digestive tract and the CNS (Mayer, 2011). This concept was further extended by microbiota-focused research demonstrating that gut microorganisms influence brain function and behavior through diverse signaling routes (Mayer, 2011; Cryan and Dinan, 2012). Within this integrated system, vagal afferent signaling conveys information about the physiological state of the gut—from nutrient availability to microbial balance—to the NTS and downstream brainstem, hypothalamic, and limbic networks, where it is integrated and transformed into adaptive autonomic, metabolic, and behavioral responses (Mayer, 2011; Cryan and Dinan, 2012; Sharon et al., 2016). A key advancement in this field is the identification of cellular structures enabling rapid gut-to-brain signaling, which has transformed the brain-gut axis from an abstract systems concept into a defined neuroepithelial signaling pathway (Kaelberer et al., 2018). Recent studies have highlighted the importance of epithelial–neuronal communication in gut–brain signaling. It was initially proposed that sensory enteroendocrine cells (EECs), through specialized basal processes termed neuropods, establish direct synapse-like communication with vagal afferents, enabling rapid glutamatergic transmission of luminal signals (Bellono et al., 2017; Kaelberer et al., 2018). However, although this hypothesis was supported by functional studies, direct morphological evidence demonstrating bona fide synaptic contacts *in vivo* has remained lacking. More recent anatomical studies from multiple independent laboratories have consistently shown that enteroendocrine cells, including enterochromaffin and L cells, rarely or never form synapse-like contacts with vagal or spinal afferent endings in the gut mucosa, and that most cells are separated by distances incompatible with conventional synapse formation (Spencer et al., 2024a,b; Cao et al., 2024). Consistent with these findings, recent work similarly concluded that while a subset of EECs is closely juxtaposed to sensory nerve fibers, the majority are too distant to establish bona fide synapses and are therefore more likely to communicate through paracrine signaling (Touhara et al., 2025). Thus, although epithelial–neuronal communication is recognized as an important component of gut–brain signaling, current evidence suggests that this communication predominantly occurs through paracrine mechanisms rather than direct synaptic transmission, while the physiological significance of occasional close appositions remains to be further clarified (Figure 3).

Furthermore, the microbiota dimension has added a critical layer to the brain-gut axis, influencing brain function and behavior through neural, endocrine, and immune pathways, with the vagus nerve representing a primary neural route (Cryan and Dinan, 2012; Sharon et al., 2016). Experimental study in mice showed that the ingestion of specific strains, such as Lactobacillus, alters central GABA receptor expression and reduces anxiety- and depression-like behaviors; notably, these effects are abolished by vagotomy (Bravo et al., 2011). Although these findings demonstrate vagal dependence in this experimental model, they do not exclude parallel endocrine, immune, or metabolic pathways through which microbiota alterations may influence brain function and behavior (Cryan and Dinan, 2012; Sharon et al., 2016)0.7 While primarily preclinical, this confirms that certain microbiota-driven behavioral changes are vagus nerve-dependent. Recent nutrient-circuit studies reinforce this by demonstrating that the gut biases central preference and behavior through discrete vagal pathways responsive to sugar and fat (Bravo et al., 2011; Kaelberer et al., 2018; Tan et al., 2020). Peripheral interoceptive signals also influence affective regulation beyond the gastrointestinal system. Experimentally induced tachycardia enhances anxiety-like behavior via central interoceptive processing involving the posterior insular cortex, illustrating how peripheral physiological states shape emotional responses (Hsueh et al., 2023). Collectively, vagal afferent pathways provide a major neural route through which nutrient, epithelial, immune, and microbial signals reach the brain. Although signal encoding and modulation may occur at peripheral sensory endings and within vagal sensory neurons, their integration into metabolic and behavioral outputs is thought to emerge primarily within downstream NTS–hypothalamic–limbic networks. This integrated signaling links metabolism with mental health, as inflammatory and metabolic changes in the gut acquire behavioral significance via the vagus nerve (Dragstedt, 1945; Browning and Travagli, 2010; Mayer, 2011; Cryan and Dinan, 2012; Sharon et al., 2016). Consequently, vagal dysfunction may underpin a clinical continuum where altered gut sensing, chronic inflammation, and metabolic imbalance intersect with affective symptoms. This explains why gastrointestinal physiological shifts reverberate into appetite, mood, and cognition, establishing the vagus nerve as the central interface in contemporary gut-brain models.

More broadly, vagal regulation follows a conserved peripheral afferent–brainstem–efferent framework, while organ-specific circuits adapt this organization to the rapid reflex control of cardiovascular function and the broader endocrine, immune, metabolic, and behavioral dynamics of gastrointestinal physiology. This shared yet functionally specialized organization may also contribute to variability in VNS outcomes and the difficulty of selectively targeting specific cardiovascular or metabolic functions.

## 4 Discussion

### 4.1 Vagus nerve stimulation (VNS) intervention mechanism

The anatomical integration of the vagus nerve into the autonomic nervous system provides a robust foundation for VNS, a neuromodulatory technique designed to regulate systemic homeostasis (Badran and Austelle, 2022; Bu et al., 2026). The primary rationale for VNS centers on the accessibility of the cervical vagus nerve, which allows for targeted modulation of visceral functions (Badran and Austelle, 2022; Bu et al., 2026). While invasive VNS (iVNS), transcutaneous auricular VNS (taVNS), and transcutaneous cervical VNS (tcVNS) utilize distinct anatomical interfaces—ranging from direct neural wrapping to cutaneous stimulation—they share a fundamental neurophysiological mechanism (Badran and Austelle, 2022; Bu et al., 2026). Each modality activates peripheral afferent fibers that ultimately converge at the NTS in the medulla oblongata (Badran and Austelle, 2022; Bu et al., 2026). This shared sensory gateway allows diverse methods to modulate central autonomic networks and inflammatory responses via distinct procedural methodologies ranging from surgical implantation to transcutaneous application. The regulatory landscape has evolved from the Food and Drug Administration (FDA) early approvals for epilepsy (1997) and depression (2005) to recent clearances for non-invasive devices of headaches (Afra et al., 2021; Badran and Austelle, 2022). Modern clinical applications now include stroke rehabilitation and rheumatoid arthritis (Badran and Austelle, 2022; Liu et al., 2022; Tesser et al., 2026).

A critical objective of VNS is the restoration of autonomic equilibrium by addressing the sympathetic hyperactivity and parasympathetic withdrawal characteristic of chronic diseases (Bu et al., 2026). Cervical stimulation facilitates this through a dual pathway. The efferent pathway enhances parasympathetic activity, providing inhibitory braking signals to the heart and viscera to reduce heart rate and stabilize organ function. Simultaneously, the afferent pathway utilizes sensory fibers to trigger central reflex arcs that suppress systemic sympathetic outflow. This reciprocal modulation is essential for stabilizing heart rate variability and reducing systemic blood pressure (Bender et al., 2026; Liu et al., 2026). Furthermore, fiber recruitment is intensity-dependent; low-intensity VNS primarily activates large-diameter afferent A-fibers to modulate central sympathetic drive, whereas higher intensities are required to recruit efferent B-fibers for direct parasympathetic organ control (McAllen et al., 2018; Patros et al., 2024; Cheng et al., 2025).

Despite its potential, VNS faces critical challenges including non-standardized parameters, poor fiber selectivity, and high response variability among patients. A major unresolved limitation is the incomplete knowledge of the spatial organization of functional fiber populations within the human vagus nerve. Without detailed anatomical mapping, selective recruitment of afferent versus efferent pathways and organ-specific projections remains difficult, hindering mechanistic interpretation and limiting the development of targeted neuromodulation strategies (Bu et al., 2026; Horowitz et al., 2026). Furthermore, technical hurdles in non-invasive dosing and placebo design underscore the urgent need for a more rigorous, human-centered understanding of vagal architecture (Chen and Liu, 2025; Tousif et al., 2025; Horowitz et al., 2026).

### 4.2 Latest findings, limitations and future perspectives

Recent research on the vaugs nerve has branched into several sophisticated directions, most notably the integration of high-throughput omics technologies with traditional neuroanatomy. Current findings are increasingly focused on single-cell RNA sequencing (scRNA-seq) and spatial transcriptomics to identify the molecular signatures of vagal neurons. These omics-driven approaches aim to categorize vagal fibers by their specific genetic markers and receptor expressions in rodent, large-animal and human studies (Kupari et al., 2019; Zhao et al., 2022; Hu et al., 2025; Pan et al., 2026). Emerging studies suggest that these molecularly defined vagal populations are associated with distinct physiological functions (Kupari et al., 2019; Zhao et al., 2022). For example, genetically defined vagal sensory neurons expressing specific receptor profiles have been implicated in mechanosensation, nutrient sensing (Williams et al., 2016; Bai et al., 2019), and cardiovascular reflex regulation (Lovelace et al., 2023), whereas molecularly distinct vagal motor populations contribute to organ-specific autonomic outputs. Defining these sensory and motor identities may therefore provide a biological basis for developing more selective cell- and pathway-targeted neuromodulation strategies. This is particularly relevant to the dual roles of the nerve: in the cardiovascular system, it involves the precise monitoring of cardiac cycles and pressure changes, while in the gastrointestinal tract, it encompasses the complex sensing of gut microbiota, metabolic hormones, and the gut-brain axis (Zhao et al., 2022; Hu et al., 2025; Pan et al., 2026). By mapping these molecular landscapes, researchers seek to decipher how the brain discriminates between different visceral signals.

The vagus nerve functions as a vital chemical bridge between the brain and the cardiovascular and gastrointestinal systems, maintaining homeostatic regulation through a sophisticated neurochemical landscape (Assas et al., 2014; Prescott and Liberles, 2022; Holt, 2022). Within the the DMV, the NA, and the NTS, classical neurotransmitters such as glutamate and GABA mediate rapid synaptic transmission and autonomic reflexes, while diverse biogenic amines and neuropeptides (e.g., NO, GLP-1, PYY, ghrelin and CCK) act as critical neuromodulators for long-term physiological processes like appetite and stress responses (Harada et al., 1993; Li et al., 2001; Date et al., 2002; Waise et al., 2018; Prescott and Liberles, 2022). Findings from animal models, together with clinical observations in humans, suggest that disruptions in these neurochemical signals are associated with depression, anxiety, and neurological disorders, often manifesting as reduced heart rate variability and impaired autonomic stability (Harada et al., 1993; Waise et al., 2018; Siopi et al., 2023). Recent research has therefore focused on mapping these molecular landscapes to decipher how the brain discriminates between complex visceral signals.

These molecular studies have also raised an important question regarding the organizational principles of the vagus nerve. While recent transcriptomic and functional evidence supports the existence of organ-specific vagal populations, particularly within cardiovascular and gastrointestinal pathways, convergent processing within central autonomic nuclei suggests that vagal signaling cannot be viewed as entirely compartmentalized (Zhao et al., 2022; P. E. Sawchenko, 1983). Rather, the vagus circuits likely operate through a hierarchical organization in which specialized peripheral circuits are integrated within shared central networks. Elucidating this balance between specificity and integration is crucial for understanding cardiometabolic comorbidities and designing next-generation neuromodulation strategies that can selectively target organ-specific fibers.

However, most vagus nerve studies rely on rodent models, which fail to encapsulate the scale and complex fascicular organization of the human vagus nerve. Comparative anatomical studies have shown that the mice vagus nerve typically consists of a single fascicle, whereas the human vagus nerve contains multiple fascicles embedded within abundant connective tissue, resulting in a markedly more complex internal architecture. Although porcine vagus nerves more closely resemble human nerves in size and fascicular organization, species-specific differences remain, highlighting the need for direct human anatomical investigations (Stakenborg et al., 2020). Because we lack precise human fascicular mapping, the micro-anatomical distribution of specific fiber types within the nerve trunk remains highly uncertain across different segmental levels (Pelot et al., 2020; Thompson et al., 2026). This structural blind spot directly limits clinical applications like VNS without detailed maps, current VNS indiscriminately recruits a broad mix of fibers, triggering off-target side effects instead of organ-specific therapeutic outcomes (Chen and Liu, 2025). This precision gap is further compounded by our inability to distinguish between afferent and efferent fiber recruitment *in vivo*, obstructing the isolation of directional signaling loops within the heart-brain and gut-brain axes (Bu et al., 2026).

Additionally, despite advances in single-cell transcriptomics categorizing vagal neurons, it remains difficult to link these molecular profiles with actual structural targets like the cardiac plexus or specific intestinal layers (Zhao et al., 2022). The transgenic mice research also have clear translational limits. While these models allow cell-specific manipulations in rodents, they cannot replicate the dense, larger-scale fascicular architecture of humans (Pelot et al., 2020). Ultimately, resolving the current questions in neurovisceral physiology depends heavily on overcoming these specific methodological capabilities.

To overcome these challenges, future research should prioritize a multi-scale approach that integrates micro-anatomical human studies with sequencing data, such as transcriptomic profiles of human vagal ganglia. Quantifying essential parameters including fiber diameters, fascicular arrangements, and specific genetic markers is a prerequisite for advancing the field. Such research is necessary to address the current limitations of VNS, specifically the uncertainty regarding optimal stimulation doses and the inability to predict individual patient responses. Without a comprehensive anatomical and molecular atlas based on human cadaveric research, we cannot effectively resolve the variability in clinical outcomes. Bridging the gap between structural architecture and molecular identity might be the key to unlocking the full therapeutic capacity of the vagus nerve, potentially enabling the development of more precise, patient-specific neuromodulation strategies.

An additional unresolved question is whether molecularly and functionally distinct vagal neuronal populations exhibit differential susceptibility to cardiometabolic diseases. While disease-associated remodeling has been observed in both cardiovascular and metabolic circuits, it remains unclear whether these conditions converge on shared vagal pathways or preferentially affect specific neuronal subpopulations. Such heterogeneity may partially explain the variability in clinical responses to VNS and raises the possibility that neuromodulatory efficacy could depend on both disease context and stage of progression.

## 5 Conclusion

The vagus nerve functions as a multidimensional neurovisceral interface integrating cardiovascular, metabolic, and gut–brain signaling through complex bidirectional pathways. Rather than a simple parasympathetic effector, it operates as an integrated regulatory network linking peripheral physiology with central neural processing. Advances in neuromodulation and omics technologies have expanded its clinical and mechanistic relevance, yet significant gaps remain in human-specific anatomical and molecular understanding. Future research integrating structural and molecular insights is essential to enable precise, targeted vagal therapies and improve clinical outcomes.

## Glossary

NTS: nucleus tractus solitarius
NA: nucleus ambiguous
SA: sinoatrial
AV: atrioventricular
ICNS: intrinsic cardiac nervous system
GP: ganglionated plexi
TH: tyrosine hydroxylase
ChAT: choline acetyltransferase
RAGP: right atrial ganglionated plexus
NPY: neuropeptide Y
RVOT: right ventricular outflow tract
nNOS: neuronal nitric oxide synthase
ROS: reactive oxygen species
TRPV-1: transient receptor potential vanilloid 1
CGRP: calcitonin gene-related peptide
NMDA: n-methyl-D-aspartate
GABA: gamma-aminobutyric acid
VANs: vagal afferent neurons
CNS: central nervous system
CCK: cholecystokinin
GLP-1: glucagon-like peptide-1
PYY: peptide YY
T2DM: type 2 diabetes mellitus
EECs: sensory enteroendocrine cells
VNS: vagus nerve stimulation
iVNS: invasive vagus nerve stimulation
taVNS: transcutaneous auricular vagus nerve stimulation
tcVNS: transcutaneous cervical vagus nerve stimulation
FDA: food and drug administration
scRNA-seq: single-cell RNA sequencing

## References

[ref1] Afra P. Adamolekun B. Aydemir S. Watson G. D. R. (2021). Evolution of the vagus nerve stimulation (VNS) therapy system technology for drug-resistant epilepsy. Front. Med. Technol. 3:696543. doi: 10.3389/fmedt.2021.696543, 35047938 PMC8757869

[ref2] Ajijola O. A. Howard-Quijano K. Scovotti J. Vaseghi M. Lee C. Mahajan A. . (2015). Augmentation of cardiac sympathetic tone by percutaneous low-level stellate ganglion stimulation in humans: a feasibility study. Physiol. Rep. 3:e12328. doi: 10.14814/phy2.12328, 25804262 PMC4393162

[ref3] Altschuler S. M. Bao X. M. Bieger D. Hopkins D. A. Miselis R. R. (1989). Viscerotopic representation of the upper alimentary tract in the rat: sensory ganglia and nuclei of the solitary and spinal trigeminal tracts. J. Comp. Neurol. 283, 248–268. doi: 10.1002/cne.902830207, 2738198

[ref4] Ardell J. L. Armour J. A. (2016). Neurocardiology: structure-based function. Compr. Physiol. 6, 1635–1653. doi: 10.1002/cphy.c150046, 27783854

[ref5] Armour J. A. (2008). Potential clinical relevance of the 'little brain' on the mammalian heart. Exp. Physiol. 93, 165–176. doi: 10.1113/expphysiol.2007.041178, 17981929

[ref6] Armour J. A. Murphy D. A. Yuan B. X. Macdonald S. Hopkins D. A. (1997). Gross and microscopic anatomy of the human intrinsic cardiac nervous system. Anat. Rec. 247, 289–298. doi: 10.1002/(SICI)1097-0185(199702)247:2<289::AID-AR15>3.0.CO;2-L, 9026008

[ref7] Assas B. M. Pennock J. I. Miyan J. A. (2014). Calcitonin gene-related peptide is a key neurotransmitter in the neuro-immune axis. Front. Neurosci. 8:23. doi: 10.3389/fnins.2014.00023, 24592205 PMC3924554

[ref8] Badran B. W. Austelle C. W. (2022). The future is noninvasive: a brief review of the evolution and clinical utility of vagus nerve stimulation. Focus (Am Psychiatr Publ) 20, 3–7. doi: 10.1176/appi.focus.20210023, 35746934 PMC9063597

[ref9] Bai L. Mesgarzadeh S. Ramesh K. S. Huey E. L. Liu Y. Gray L. A. . (2019). Genetic identification of vagal sensory neurons that control feeding. Cell 179, 1129–1143.e23. doi: 10.1016/j.cell.2019.10.031, 31730854 PMC6916730

[ref10] Bellono N. W. Bayrer J. R. Leitch D. B. Castro J. Zhang C. O'Donnell T. A. . (2017). Enterochromaffin cells are gut chemosensors that couple to sensory neural pathways. Cell 170, 185–198 e116. doi: 10.1016/j.cell.2017.05.034 28648659 PMC5839326

[ref11] Bender S. A. Green D. B. Thakkar V. S. Zimmerman H. L. Elazab M. Kilgore K. L. . (2026). Single-electrode, bidirectional control of heart rate via Vagus nerve modulation in rat model. IEEE Trans. Neural Syst. Rehabil. Eng. 34, 1144–1154. doi: 10.1109/TNSRE.2026.3663907, 41671117 PMC13021142

[ref12] Berthoud H. R. (2008). The vagus nerve, food intake and obesity. Regul. Pept. 149, 15–25. doi: 10.1016/j.regpep.2007.08.024, 18482776 PMC2597723

[ref13] Berthoud H. R. Neuhuber W. L. (2000). Functional and chemical anatomy of the afferent vagal system. Auton. Neurosci. 85, 1–17. doi: 10.1016/S1566-0702(00)00215-0, 11189015

[ref14] Biscola N. P. Bartmeyer P. M. Beshay Y. Stern E. Mihaylov P. V. Powley T. L. . (2024). Laterality, sexual dimorphism, and human vagal projectome heterogeneity shape neuromodulation to vagus nerve stimulation. Commun. Biol. 7:1536. doi: 10.1038/s42003-024-07222-1, 39562711 PMC11576867

[ref15] Bonaz B. Sinniger V. Pellissier S. (2017). The vagus nerve in the neuro-immune axis: implications in the pathology of the gastrointestinal tract. Front. Immunol. 8:1452. doi: 10.3389/fimmu.2017.01452, 29163522 PMC5673632

[ref16] Bravo J. A. Forsythe P. Chew M. V. Escaravage E. Savignac H. M. Dinan T. G. . (2011). Ingestion of Lactobacillus strain regulates emotional behavior and central GABA receptor expression in a mouse via the vagus nerve. Proc. Natl. Acad. Sci. USA 108, 16050–16055. doi: 10.1073/pnas.1102999108, 21876150 PMC3179073

[ref17] Browning K. N. Travagli R. A. (2010). Plasticity of vagal brainstem circuits in the control of gastric function. Neurogastroenterol. Motil. 22, 1154–1163. doi: 10.1111/j.1365-2982.2010.01592.x, 20804520 PMC2970760

[ref18] Browning K. N. Travagli R. A. (2014). Central nervous system control of gastrointestinal motility and secretion and modulation of gastrointestinal functions. Compr. Physiol. 4, 1339–1368. doi: 10.1002/cphy.c130055 25428846 PMC4858318

[ref19] Bu Y. Liang A. Hoffman B. U. Schiehser D. M. Case O. Simmons A. . (2026). A review of vagus nerve stimulation for disease: comprehensive theory and evidence for mechanisms of action. Compr. Physiol. 16:e70109. doi: 10.1002/cph4.70109, 41781173 PMC12960021

[ref20] Cao J. M. Chen L. S. KenKnight B. H. Ohara T. Lee M. H. Tsai J. . (2000). Nerve sprouting and sudden cardiac death. Circ. Res. 86, 816–821. doi: 10.1161/01.res.86.7.816, 10764417

[ref21] Cao N. Merchant W. Gautron L. (2024). Limited evidence for anatomical contacts between intestinal GLP-1 cells and vagal neurons in male mice. Sci. Rep. 14:23666. doi: 10.1038/s41598-024-74000-8, 39390033 PMC11467209

[ref22] Chen Z. Liu K. (2025). Mechanism and applications of vagus nerve stimulation. Curr. Issues Mol. Biol. 47:122. doi: 10.3390/cimb47020122, 39996843 PMC11854789

[ref23] Cheng K. P. Deshmukh A. Gholston A. K. Settell M. L. Knudsen B. E. LaLuzerne M. . (2025). Application of kilohertz-frequency block to mitigate off-target motor effects of vagus nerve stimulation in swine. Nat. Commun. 17:1066. doi: 10.1038/s41467-025-67823-0, 41469406 PMC12852918

[ref24] Cryan J. F. Dinan T. G. (2012). Mind-altering microorganisms: the impact of the gut microbiota on brain and behaviour. Nat. Rev. Neurosci. 13, 701–712. doi: 10.1038/nrn3346, 22968153

[ref25] Date Y. Murakami N. Toshinai K. Matsukura S. Niijima A. Matsuo H. . (2002). The role of the gastric afferent vagal nerve in ghrelin-induced feeding and growth hormone secretion in rats. Gastroenterology 123, 1120–1128. doi: 10.1053/gast.2002.35954, 12360474

[ref26] de Lartigue G. (2016). Role of the vagus nerve in the development and treatment of diet-induced obesity. J. Physiol. 594, 5791–5815. doi: 10.1113/JP271538, 26959077 PMC5063945

[ref27] Debas H. T. Carvajal S. H. (1994). Vagal regulation of acid secretion and gastrin release. Yale J. Biol. Med. 67, 145–151. 7502523 PMC2588919

[ref28] Décarie-Spain L. Hayes A. M. Lauer L. T. Kanoski S. E. (2024). The gut-brain axis and cognitive control: a role for the vagus nerve. Semin. Cell Dev. Biol 156, 201–209. doi: 10.1016/j.semcdb.2023.02.004 36803834 PMC10427741

[ref29] Dockray G. J. (2009). Cholecystokinin and gut-brain signalling. Regul. Pept. 155, 6–10. doi: 10.1016/j.regpep.2009.03.015, 19345244

[ref30] Dockray G. J. (2014). Gastrointestinal hormones and the dialogue between gut and brain. J. Physiol. 592, 2927–2941. doi: 10.1113/jphysiol.2014.270850, 24566540 PMC4214649

[ref31] Dragstedt L. R. (1945). Vagotomy for gastroduodenal ulcer. Ann. Surg. 122, 973–989. doi: 10.1097/00000658-194512260-00008 21005197

[ref32] Fedele L. Brand T. (2020). The intrinsic cardiac nervous system and its role in cardiac pacemaking and conduction. J. Cardiovasc. Dev. Dis. 7:54. doi: 10.3390/jcdd7040054, 33255284 PMC7712215

[ref33] Furness J. B. (2012). The enteric nervous system and neurogastroenterology. Nat. Rev. Gastroenterol. Hepatol. 9, 286–294. doi: 10.1038/nrgastro.2012.32, 22392290

[ref34] Grill H. J. Hayes M. R. (2012). Hindbrain neurons as an essential hub in the neuroanatomically distributed control of energy balance. Cell Metab. 16, 296–309. doi: 10.1016/j.cmet.2012.06.015, 22902836 PMC4862653

[ref35] Gumpangseth T. Mahakkanukrauh P. Das S. Cho K. H. Kim J. H. Murakami G. . (2019). Nerve distribution in myocardium including the atrial and ventricular septa in late stage human fetuses. Anat. Cell Biol. 52, 48–56. doi: 10.5115/acb.2019.52.1.48 30984452 PMC6449578

[ref36] Han W. Tellez L. A. Perkins M. H. Perez I. O. Qu T. Ferreira J. . (2018). A neural circuit for gut-induced reward. Cell 175, 665–678.e23. doi: 10.1016/j.cell.2018.08.049 30245012 PMC6195474

[ref37] Hanna P. Dacey M. J. Brennan J. Moss A. Robbins S. Achanta S. . (2021). Innervation and neuronal control of the mammalian sinoatrial node a comprehensive atlas. Circ. Res. 128, 1279–1296. doi: 10.1161/CIRCRESAHA.120.318458, 33629877 PMC8284939

[ref38] Hanna P. Rajendran P. S. Ajijola O. A. Vaseghi M. Armour J. A. Ardell J. L. . (2017). Cardiac neuroanatomy-imaging nerves to define functional control. Auton. Neurosci. 207, 48–58. doi: 10.1016/j.autneu.2017.07.008, 28802636 PMC5680093

[ref39] Harada S. Tokunaga S. Momohara M. Masaki H. Tagawa T. Imaizumi T. . (1993). Inhibition of nitric oxide formation in the nucleus tractus solitarius increases renal sympathetic nerve activity in rabbits. Circ. Res. 72, 511–516. doi: 10.1161/01.res.72.3.511, 8431981

[ref40] Holt M. K. (2022). The ins and outs of the caudal nucleus of the solitary tract: an overview of cellular populations and anatomical connections. J. Neuroendocrinol. 34:e13132. doi: 10.1111/jne.13132, 35509189 PMC9286632

[ref41] Hoover D. B. Ganote C. E. Ferguson S. M. Blakely R. D. Parsons R. L. (2004). Localization of cholinergic innervation in guinea pig heart by immunohistochemistry for high-affinity choline transporters. Cardiovasc. Res. 62, 112–121. doi: 10.1016/j.cardiores.2004.01.012, 15023558

[ref42] Horowitz M. A. Sussman J. H. Zomalan B. Rendler J. Singh A. Birouty N. . (2026). Vagus nerve stimulation: an update of currently registered clinical trials on ClinicalTrials.gov. Surg. Neurol. Int. 17:64. doi: 10.25259/SNI_771_2025, 41783176 PMC12954253

[ref43] Hsueh B. Chen R. Jo Y. Tang D. Raffiee M. Kim Y. S. . (2023). Cardiogenic control of affective behavioural state. Nature 615, 292–299. doi: 10.1038/s41586-023-05748-8, 36859543 PMC9995271

[ref44] Hu Y. Dai S. Chen L. Ma X. Li H. Lu Y. . (2025). Multi-omics reveals the mechanism of vagus nerve stimulation in the treatment of chronic congestive heart failure. Sci. Rep. 15:19613. doi: 10.1038/s41598-025-04397-3, 40467772 PMC12137920

[ref45] Jänig W. (2022). The Integrative Action of the Autonomic Nervous system: Neurobiology of Homeostasis. Cambridge: Cambridge University Press.

[ref46] Kaelberer M. M. Buchanan K. L. Klein M. E. Barth B. B. Montoya M. M. Shen X. . (2018). A gut-brain neural circuit for nutrient sensory transduction. Science 361:eaat5236. doi: 10.1126/science.aat5236, 30237325 PMC6417812

[ref47] Kalia M. Sullivan J. M. (1982). Brainstem projections of sensory and motor components of the vagus nerve in the rat. J. Comp. Neurol. 211, 248–264. doi: 10.1002/cne.902110304, 7174893

[ref48] Katritsis D. G. Pokushalov E. Romanov A. Giazitzoglou E. Siontis G. C. Po S. S. . (2013). Autonomic denervation added to pulmonary vein isolation for paroxysmal atrial fibrillation: a randomized clinical trial. J. Am. Coll. Cardiol. 62, 2318–2325. doi: 10.1016/j.jacc.2013.06.053, 23973694

[ref49] Kawano H. Okada R. Yano K. (2003). Histological study on the distribution of autonomic nerves in the human heart. Heart Vessel. 18, 32–39. doi: 10.1007/s003800300005, 12644879

[ref50] Kupari J. Haring M. Agirre E. Castelo-Branco G. Ernfors P. (2019). An atlas of vagal sensory neurons and their molecular specialization. Cell Rep. 27, 2508–2523.e4. doi: 10.1016/j.celrep.2019.04.096, 31116992 PMC6533201

[ref51] Li D. P. Averill D. B. Pan H. L. (2001). Differential roles for glutamate receptor subtypes within commissural NTS in cardiac-sympathetic reflex. Am. J. Physiol. Regul. Integr. Comp. Physiol. 281, R935–R943. doi: 10.1152/ajpregu.2001.281.3.R935, 11507011

[ref52] Liu Z. Lu S. Haskell I. A. Schappe M. S. Josipovic M. Min S. . (2026). Vagal blood volume receptors compensate for haemorrhage and posture change. Nature 651, 1068–1076. doi: 10.1038/s41586-025-10010-4, 41606321 PMC13017543

[ref53] Liu C. Y. Russin J. Adelson D. P. Jenkins A. Hilmi O. Brown B. . (2022). Vagus nerve stimulation paired with rehabilitation for stroke: implantation experience from the VNS-REHAB trial. J. Clin. Neurosci. 105, 122–128. doi: 10.1016/j.jocn.2022.09.013, 36182812

[ref54] Longo S. Rizza S. Federici M. (2023). Microbiota-gut-brain axis: relationships among the vagus nerve, gut microbiota, obesity, and diabetes. Acta Diabetol. 60, 1007–1017. doi: 10.1007/s00592-023-02088-x, 37058160 PMC10289935

[ref55] Lovelace J. W. Ma J. Yadav S. Chhabria K. Shen H. Pang Z. . (2023). Vagal sensory neurons mediate the Bezold-Jarisch reflex and induce syncope. Nature 623, 387–396. doi: 10.1038/s41586-023-06680-7, 37914931 PMC10632149

[ref56] Malliani A. (2000). Principles of Cardiovascular Neural Regulation in Health and Disease. Massachusetts, MA: Springer Science & Business Media.

[ref57] Mayer E. A. (2011). Gut feelings: the emerging biology of gut-brain communication. Nat. Rev. Neurosci. 12, 453–466. doi: 10.1038/nrn3071, 21750565 PMC3845678

[ref58] McAllen R. M. Shafton A. D. Bratton B. O. Trevaks D. Furness J. B. (2018). Calibration of thresholds for functional engagement of vagal a, B and C fiber groups in vivo. Bioelectron. Med. (Lond) 1, 21–27. doi: 10.2217/bem-2017-0001, 29480903 PMC5811083

[ref60] Min S. Chang R. B. Prescott S. L. Beeler B. Joshi N. R. Strochlic D. E. . (2019). Arterial baroreceptors sense blood pressure through decorated aortic claws. Cell Rep. 29, 2192–2201.e3. doi: 10.1016/j.celrep.2019.10.040, 31747594 PMC6893869

[ref61] Minisi A. J. Thames M. D. (1991). Activation of cardiac sympathetic afferents during coronary occlusion. Evidence for reflex activation of sympathetic nervous system during transmural myocardial ischemia in the dog. Circulation 84, 357–367. doi: 10.1161/01.cir.84.1.357, 2060106

[ref62] Mohanta S. K. Yin C. Weber C. Godinho-Silva C. Veiga-Fernandes H. Xu Q. J. . (2023). Cardiovascular brain circuits. Circ. Res. 132, 1546–1565. doi: 10.1161/CIRCRESAHA.123.322791, 37228235 PMC10231443

[ref63] Murphy K. G. Bloom S. R. (2006). Gut hormones and the regulation of energy homeostasis. Nature 444, 854–859. doi: 10.1038/nature05484, 17167473

[ref64] Nerantzis C. E. Papachristos J. C. Gribizi J. E. Voudris V. A. Infantis G. P. Koroxenidis G. T. (1996). Functional dominance of the right coronary artery: incidence in the human heart. Clin. Anat. 9, 10–13. doi: 10.1002/(SICI)1098-2353(1996)9:1<10::AID-CA2>3.0.CO;2-3, 8838273

[ref001] Nieuwenhuys R. Voogd J. Van Huijzen C. (2008). The Human Central Nervous System. Heidelberg: Springer Berlin Heidelberg.

[ref65] Palma J. A. Benarroch E. E. (2014). Neural control of the heart: recent concepts and clinical correlations. Neurology 83, 261–271. doi: 10.1212/WNL.0000000000000605, 24928126

[ref66] Pan D. Jiang M. Wang Y. He J. Tang J. Liu S. . (2026). Multi-omics reveals associations between the microbiota-gut-brain axis and antidepressant effects of vagus nerve stimulation. Neurobiol. Stress 40:100777. doi: 10.1016/j.ynstr.2025.100777, 41492358 PMC12765250

[ref67] Patros M. Farmer D. G. S. Moneghetti K. Ottaviani M. M. Sivathamboo S. Simpson H. D. . (2024). First-in-human microelectrode recordings from the vagus nerve during clinical vagus nerve stimulation. Epilepsia Open 9, 2522–2527. doi: 10.1002/epi4.13083, 39465627 PMC11633718

[ref68] Pauza D. H. Skripka V. Pauziene N. (2002). Morphology of the intrinsic cardiac nervous system in the dog: a whole-mount study employing histochemical staining with acetylcholinesterase. Cells Tissues Organs 172, 297–320. doi: 10.1159/000067198, 12566631

[ref69] Pavlov V. A. Tracey K. J. (2012). The vagus nerve and the inflammatory reflex--linking immunity and metabolism. Nat. Rev. Endocrinol. 8, 743–754. doi: 10.1038/nrendo.2012.189, 23169440 PMC4082307

[ref70] Pelot N. A. Goldhagen G. B. Cariello J. E. Musselman E. D. Clissold K. A. Ezzell J. A. . (2020). Quantified morphology of the cervical and subdiaphragmatic Vagus nerves of human, pig, and rat. Front. Neurosci. 14:601479. doi: 10.3389/fnins.2020.601479, 33250710 PMC7672126

[ref71] Prescott S. L. Liberles S. D. (2022). Internal senses of the vagus nerve. Neuron 110, 579–599. doi: 10.1016/j.neuron.2021.12.020, 35051375 PMC8857038

[ref72] Salavatian S. Hoang J. D. Yamaguchi N. Lokhandwala Z. A. Swid M. A. Armour J. A. . (2022). Myocardial infarction reduces cardiac nociceptive neurotransmission through the vagal ganglia. JCI Insight 7:e155747. doi: 10.1172/jci.insight.155747, 35015733 PMC8876456

[ref73] Saper C. B. (2002). The central autonomic nervous system: conscious visceral perception and autonomic pattern generation. Annu. Rev. Neurosci. 25, 433–469. doi: 10.1146/annurev.neuro.25.032502.111311, 12052916

[ref74] Sawchenko P. E. (1983). Central connections of the sensory and motor nuclei of the vagus nerve. J. Auton. Nerv. Syst. 9, 13–26. doi: 10.1016/0165-1838(83)90129-7, 6319474

[ref75] Schirmer B. D. (1989). Current status of proximal gastric vagotomy. Ann. Surg. 209, 131–148. doi: 10.1097/00000658-198902000-00001, 2644897 PMC1493911

[ref76] Schwartz P. J. Vanoli E. Stramba-Badiale M. De Ferrari G. M. Billman G. E. Foreman R. D. (1988). Autonomic mechanisms and sudden death. New insights from analysis of baroreceptor reflexes in conscious dogs with and without a myocardial infarction. Circulation 78, 969–979. doi: 10.1161/01.cir.78.4.969, 3168199

[ref77] Settell M. L. Pelot N. A. Knudsen B. E. Dingle A. M. McConico A. L. Nicolai E. N. . (2020). Functional vagotopy in the cervical vagus nerve of the domestic pig: implications for the study of vagus nerve stimulation. J. Neural Eng. 17:026022. doi: 10.1088/1741-2552/ab7ad4, 32108590 PMC7306215

[ref78] Sharma B. Jones K. A. Lober R. M. Hatcher-Solis C. N. (2025). Left and right vagus nerve stimulation: historical perspectives, clinical efficacy, and future directions. Front. Hum. Neurosci. 19:1609654. doi: 10.3389/fnhum.2025.1609654, 40799471 PMC12339557

[ref79] Sharon G. Sampson T. R. Geschwind D. H. Mazmanian S. K. (2016). The central nervous system and the gut microbiome. Cell 167, 915–932. doi: 10.1016/j.cell.2016.10.027, 27814521 PMC5127403

[ref80] Shenton F. C. Campbell T. Jones J. F. X. Pyner S. (2021). Distribution and morphology of sensory and autonomic fibres in the subendocardial plexus of the rat heart. J. Anat. 238, 36–52. doi: 10.1111/joa.13284, 32783212 PMC7754995

[ref81] Siopi E. Galerne M. Rivagorda M. Saha S. Moigneu C. Moriceau S. . (2023). Gut microbiota changes require vagus nerve integrity to promote depressive-like behaviors in mice. Mol. Psychiatry 28, 3002–3012. doi: 10.1038/s41380-023-02071-6, 37131071 PMC10615761

[ref82] Song X. J. Zhao Z. Q. (1993). Differential effects of NMDA and non-NMDA receptor antagonists on spinal cutaneous vs muscular nociception in the cat. Neuroreport 4, 17–20. doi: 10.1097/00001756-199301000-00004, 8095819

[ref83] Spencer N. J. Kyloh M. A. Travis L. Hibberd T. J. (2024a). Identification of vagal afferent nerve endings in the mouse colon and their spatial relationship with enterochromaffin cells. Cell Tissue Res. 396, 313–327. doi: 10.1007/s00441-024-03879-6, 38383905 PMC11144134

[ref84] Spencer N. J. Kyloh M. A. Travis L. Hibberd T. J. (2024b). Mechanisms underlying the gut-brain communication: how enterochromaffin (EC) cells activate vagal afferent nerve endings in the small intestine. J. Comp. Neurol. 532:e25613. doi: 10.1002/cne.25613, 38625817

[ref85] Stakenborg N. Gomez-Pinilla P. J. Verlinden T. J. M. Wolthuis A. M. D'Hoore A. Farre R. . (2020). Comparison between the cervical and abdominal vagus nerves in mice, pigs, and humans. Neurogastroenterol. Motil. 32:e13889. doi: 10.1111/nmo.13889 32476229 PMC7507132

[ref86] Standring S. (2021). Gray's Anatomy: the Anatomical Basis of Clinical Practice. Philadephia, PA: Elsevier.

[ref87] Tack J. Talley N. J. Camilleri M. Holtmann G. Hu P. Malagelada J. R. . (2006). Functional gastroduodenal disorders. Gastroenterology 130, 1466–1479. doi: 10.1053/j.gastro.2005.11.059, 16678560

[ref88] Tan H. E. Sisti A. C. Jin H. Vignovich M. Villavicencio M. Tsang K. S. . (2020). The gut-brain axis mediates sugar preference. Nature 580, 511–516. doi: 10.1038/s41586-020-2199-7, 32322067 PMC7185044

[ref89] Tesser J. R. P. Crowley A. R. Box E. J. June J. P. Wickersham P. B. Valenzuela G. J. . (2026). Vagus nerve-mediated neuroimmune modulation for rheumatoid arthritis: a pivotal randomized controlled trial. Nat. Med. 32, 369–378. doi: 10.1038/s41591-025-04114-7, 41429981 PMC12823386

[ref90] Thayer J. F. Koenig J. (2025) The Neurobiology of Self-Regulation: a Neurovisceral Integration Perspective. Washington, DC, USA: American Psycological Association.

[ref91] Thompson N. Mastitskaya S. Iacoviello F. Turhani F. Shearing P. R. Aristovich K. . (2026). Human vagus nerve fascicular anatomy and its implications for targeted cardiac stimulation: a microCT segmentation and histological pilot anatomical study. Front. Neurosci. 20:1731234. doi: 10.3389/fnins.2026.1731234, 41788551 PMC12956647

[ref92] Thorén P. (2005). Role of cardiac vagal C-fibers in cardiovascular control. Rev. Physiol. Biochem. Pharmacol. 86, 1–94. doi: 10.1007/bfb0031531, 386467

[ref93] Tompkins J. D. Hoover D. B. Havton L. A. Patel J. C. Cho Y. Smith E. H. . (2025). Comparative specialization of intrinsic cardiac neurons in humans, mice and pigs. J. Physiol. 603, 2043–2070. doi: 10.1113/JP286714, 39513933 PMC11957936

[ref94] Touhara K. K. Rossen N. D. Deng F. Castro J. Harrington A. M. Chu T. . (2025). Topological segregation of stress sensors along the gut crypt-villus axis. Nature 640, 732–742. doi: 10.1038/s41586-024-08581-9, 39939779 PMC12090882

[ref95] Tousif K. Ali M. Saleem S. Raza A. Imran S. Haroon M. . (2025). Non-invasive vagus nerve stimulation for the treatment of neurological & psychiatric disorders: a narrative review. Explor. Res. Hypothesis Med 10, 205–213. doi: 10.14218/erhm.2025.00015, 42386300

[ref96] Tracey K. J. (2002). The inflammatory reflex. Nature 420, 853–859. doi: 10.1038/nature01321, 12490958

[ref97] Travagli R. A. Anselmi L. (2016). Vagal neurocircuitry and its influence on gastric motility. Nat. Rev. Gastroenterol. Hepatol. 13, 389–401. doi: 10.1038/nrgastro.2016.76, 27251213 PMC5605144

[ref98] Travagli R. A. Hermann G. E. Browning K. N. Rogers R. C. (2006). Brainstem circuits regulating gastric function. Annu. Rev. Physiol. 68, 279–305. doi: 10.1146/annurev.physiol.68.040504.094635, 16460274 PMC3062484

[ref99] Van Oudenhove L. Levy R. L. Crowell M. D. Drossman D. A. Halpert A. D. Keefer L. . (2016). Biopsychosocial aspects of functional gastrointestinal disorders: how central and environmental processes contribute to the development and expression of functional gastrointestinal disorders. Gastroenterology 150, 1355–1367.e2. doi: 10.1053/j.gastro.2016.02.027, 27144624 PMC8809487

[ref100] van Weperen V. Y. H. Vaseghi M. (2023). Cardiac vagal afferent neurotransmission in health and disease: review and knowledge gaps. Front. Neurosci. 17:1192188. doi: 10.3389/fnins.2023.1192188, 37351426 PMC10282187

[ref101] Wachsmuth H. R. Weninger S. N. Duca F. A. (2022). Role of the gut-brain axis in energy and glucose metabolism. Exp. Mol. Med. 54, 377–392. doi: 10.1038/s12276-021-00677-w, 35474341 PMC9076644

[ref102] Waise T. M. Z. Dranse H. J. Lam T. K. T. (2018). The metabolic role of vagal afferent innervation. Nat. Rev. Gastroenterol. Hepatol. 15, 625–636. doi: 10.1038/s41575-018-0062-1, 30185916

[ref103] Williams E. K. Chang R. B. Strochlic D. E. Umans B. D. Lowell B. B. Liberles S. D. (2016). Sensory neurons that detect stretch and nutrients in the digestive system. Cell 166, 209–221. doi: 10.1016/j.cell.2016.05.011, 27238020 PMC4930427

[ref104] Yamakawa K. So E. L. Rajendran P. S. Hoang J. D. Makkar N. Mahajan A. . (2014). Electrophysiological effects of right and left vagal nerve stimulation on the ventricular myocardium. Am. J. Physiol. Heart Circ. Physiol. 307, H722–H731. doi: 10.1152/ajpheart.00279.2014, 25015962 PMC4187397

[ref105] Zandstra T. E. Notenboom R. G. E. Wink J. Kies P. Vliegen H. W. Egorova A. D. . (2021). Asymmetry and heterogeneity: part and parcel in cardiac autonomic innervation and function. Front. Physiol. 12:665298. doi: 10.3389/fphys.2021.665298, 34603069 PMC8481575

[ref106] Zeng W. Z. Marshall K. L. Min S. Daou I. Chapleau M. W. Abboud F. M. . (2018). PIEZOs mediate neuronal sensing of blood pressure and the baroreceptor reflex. Science 362, 464–467. doi: 10.1126/science.aau6324, 30361375 PMC6563913

[ref107] Zhang D. Liu J. Tu H. Muelleman R. L. Cornish K. G. Li Y. L. (2014). In vivo transfection of manganese superoxide dismutase gene or nuclear factor kappaB shRNA in nodose ganglia improves aortic baroreceptor function in heart failure rats. Hypertension 63, 88–95. doi: 10.1161/HYPERTENSIONAHA.113.02057, 24101667 PMC3893036

[ref108] Zhao Q. Yu C. D. Wang R. Xu Q. J. Dai Pra R. Zhang L. . (2022). A multidimensional coding architecture of the vagal interoceptive system. Nature 603, 878–884. doi: 10.1038/s41586-022-04515-5, 35296859 PMC8967724

[ref109] Zhuo H. Ichikawa H. Helke C. J. (1997). Neurochemistry of the nodose ganglion. Prog. Neurobiol. 52, 79–107. doi: 10.1016/s0301-0082(97)00003-8, 9185234

